# Alternation in Effective Connectivity With Cognitive Aging: A Longitudinal Study of Elderly Populations

**DOI:** 10.3389/fnagi.2021.755931

**Published:** 2021-11-12

**Authors:** Xingxing Cao, Tao Liu, Jiyang Jiang, Hao Liu, Jing Zhang, Nicole A. Kochan, Haijun Niu, Henry Brodaty, Perminder S. Sachdev, Wei Wen

**Affiliations:** ^1^Key Laboratory of Biomechanics and Mechanobiology, Ministry of Education, Beijing Advanced Innovation Center for Biomedical Engineering, School of Biological Science and Medical Engineering, Beihang University, Beijing, China; ^2^Beijing Advanced Innovation Center for Big Data-Based Precision Medicine, Beijing, China; ^3^Centre for Healthy Brain Ageing, School of Psychiatry, University of New South Wales, Sydney, NSW, Australia; ^4^Neuropsychiatric Institute, Prince of Wales Hospital, Sydney, NSW, Australia; ^5^Dementia Collaborative Research Centre, University of New South Wales, Sydney, NSW, Australia

**Keywords:** cognitive aging, fMRI, effective connectivity, executive function, dynamic causal modeling

## Abstract

In this research, we investigated the alterations in the directionality and strength of regional interactions within functionally changed brain networks and their relationship to cognitive decline during the aging process in normal elderly individuals. Thirty-seven cognitively normal elderly people received resting-state fMRI scans and cognitive assessments at baseline (age = 78.65 ± 3.56 years) and at 4-year follow-up. Functional connectivity analyses were used to identify networks containing brain regions whose functions changed with age as regions of interest. The spectral dynamic causal modeling (spDCM) method was used to estimate the causal interactions within networks in subjects at different time points and in subjects with different cognitive levels to explore the alterations with cognitive aging. The results showed that, at both time points, all the networks, except the frontal-parietal network (FPN) at baseline, had mutual interactions between each pair of nodes. Furthermore, when the subjects were divided with global cognition level, lost connections were only found in the subgroup with better performance. These indicated that elderly people appeared to need more interaction pathways between brain areas with cognitive decline. We also observed that the strength of the flow of information from the left angular gyrus to the precuneus, which is associated with activation of memory retrieval and the functional hub involved in various cognitive domains, was predictive of declines in executive function with the aging process, making it a potential predictor of such situation.

## Introduction

Normal aging is accompanied by cognitive declines in various domains, including processing speed and executive function (Hedden and Gabrieli, 2004; Fjell et al., 2015). Although the underlying mechanism is not yet clear, alterations in functional connectivity between brain areas and extrinsic interactions between functional networks have been proven to play an important role in this process (Onoda et al., 2012). For example, a longitudinal study found that inter-network functional connectivity between the executive control network and the default mode network (DMN) showed a U-shaped trajectory, which means the functional segregation initially increased and later decreased with aging (Ng et al., 2016). And the longitudinal decrease in connectivity within DMN and increase in functional integration between DMN and executive control network is related to lower processing speed (Ng et al., 2016; Staffaroni et al., 2018). It is widely implicated that aging impairs resting-state functional connectivity via loss of white matter integrity, dopaminergic deficits, and amyloid deposition, thus the emergence of cognitive decline (Ferreira and Busatto, 2013).

While functional connectivity describes the correlation between areas, effective connectivity further explores the causal flows of information with directionality (Friston, 1994). With the development of the spectral dynamic causal modeling (spDCM) method (Friston et al., 2014) to model effective connectivity based on resting-state fMRI, recent studies have demonstrated the importance of abnormal flows of information in brain function-related disorders (Battistella and Simonyan, 2019; Warren et al., 2019) and cognitive aging. Two cross-sectional task-state fMRI studies on the elderly found that working memory is related to the decrease in the strength of effective connection (i.e., from the dorsal prefrontal cortex to inferior parietal lobe) which it modulate s (Heinzel et al., 2017; Hinault et al., 2019). Also, aging is accompanied by changes in the bidirectional interactions of lexical semantic expression-related brain regions, which may indicate a neural compensation with degenerating language function (Hoyau et al., 2018). However, longitudinal studies are still needed to understand how the effective connectivity in resting-state networks changes with cognitive decline during the aging process.

Previous studies have replicated findings of aging-related functional changes in the precuneus, posterior cingulate cortex, medial frontal cortex, inferior parietal cortex and supplementary motor area (SMA) (Bergfield et al., 2010; Wu et al., 2011; Li et al., 2020). In this study, based on the data-driven functional connectivity analyses, we chose the DMN, the frontal-parietal network (FPN) and the sensorimotor network (SMN) as networks of interest, which include most of the aforementioned brain areas and have previously been investigated in similar studies (Bergfield et al., 2010; Allen et al., 2011; Wu et al., 2011; Tomasi and Volkow, 2012).

**FIGURE 1:**
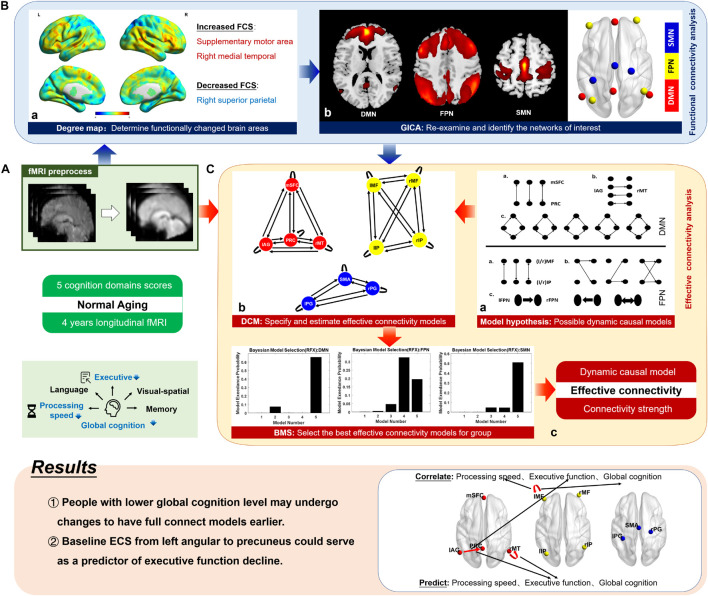
Schematic of the method. Based on the 4-year longitudinal fMRI data and five cognitive domain scores of 37 cognitively normal elderly people, we explored the alterations in the dynamic causal model structure (information propagation pathways) and effective connectivity strength (causal interaction strength) during the aging process. Supplemented by functional connectivity analysis **(B)**, this study mainly focused on effective connectivity analysis **(C)**, and its relationship with cognitive decline was further investigated (shown in results). **(A)** The raw fMRI data were preprocessed, including slice-timing correction, realignment, normalization, smoothing, and bandpass filtering. **(B)** a. We generated a degree map, which represents the voxel-based functional connectivity for each participant and compared the group-level differences between the two time points. According to family-wise error-corrected results, areas whose functional connectivity strength (FCS) significantly changed were listed. The figure shows uncorrected results, from which we can see the patterns of FCS alteration in the whole brain. b. We identified the brain networks to which significantly changed areas belong using temporal concatenation group independent component analysis (GICA). The three networks of interest were the default mode network (DMN), the fronto-parietal network (FPN), and the somatomotor network (SMN). A paired t-test was employed on the spatial maps of three networks and functional connectivity changes inner networks were found again. The spatial maps were then thresholded into clusters, and the networks were simplified into regions centering on the peak locations in the GICA maps at the group level. **(C)** a. We formulated a hypothesis for possible causal interaction model structures for the DMN and FPN. For the SMN, all mathematically possible models were considered. This provided a reasonable model space for dynamic causal modeling. b. Based on the preprocessed fMRI data, using coordinates from functional connectivity analysis, we applied the dynamic causal modeling method over predefined model space to specify and estimate the effective connectivity models. c. We then used the Bayesian model selection method to select the optimal model structure for the group by investigating how interactions at the neuronal level may have generated the observed data. The figure shows the simplified model selection result, and the optimal model was obtained using model exceedance probability. mSFC, medial superior frontal cortex; PRC, precuneus; lAG, left angular gyrus; rMT, right middle temporal gyrus; l/rMF, left/right middle frontal gyrus; l/rIP, left/right inferior parietal gyrus; SMA, supplementary motor area; l/rPG, left/right postcentral gyrus.

To examine the patterns of changes in effective connectivity in the aging brain, we performed spDCM analysis of resting-state fMRI in 37 cognitively normal elderly people in a 4-year longitudinal study. To identify the networks that functionally change with aging and locate their coordinates, the networks modeled in this study were selected based on whole-brain degree map contrasts and independent components analyses. We performed Bayesian model selection (BMS) (Penny et al., 2010) to study the patterns of alterations in interaction direction. We further examined the correlation between effective connection strength and cognition to explore the role of information flow in cognitive decline with aging (Figure 1). We hypothesized that normal elderly people have more within-network interaction pathways to perform their functions with aging, and the effective connectivity strength within networks is predictive of cognitive decline.

## Materials and Methods

### Participants

Forty cognitively normal elderly people who had functional MRI scans at two time points were drawn initially from the Sydney Memory and Ageing Study (MAS) (Sachdev et al., 2010). This is a longitudinal study of non-demented, community-dwelling individuals aged 70–90 years at baseline. At baseline, each of the 1037 MAS participants was administered a comprehensive neuropsychological test battery, and 542 also underwent an MRI scan. Individuals were excluded if they had left-handedness, a Mini-Mental State Examination (MMSE) score < 24, a diagnosis of dementia, intellectual disability, psychotic disorder, multiple sclerosis, motor neuron disease, or progressive malignancy, or could not complete the assessments due to inadequate understanding of English. All the participants were first examined in 2005–2007, and fMRI scans were performed in a proportion of the participants at 2 years (*N* = 157) and 6 years (*N* = 258) later. For the current study, we used the baseline and 4-year follow-up assessment time points. The details of the sampling methodology have been published previously (Sachdev et al., 2010).

We included participants classified as cognitively unimpaired based on neuropsychological assessments and received resting-state fMRI scans at both baseline and the 4-year follow-up (*N* = 41). After excluding one participant with imaging artifacts and corrupted images, 40 participants were included in the current study before further screening in image preprocessing.

### Neuropsychological Assessment

All the eligible participants underwent a series of comprehensive neuropsychological battery assessments (Sachdev et al., 2010). Five cognitive domains were tested: processing speed, executive function, language, visuo-spatial, and memory. The neuropsychological assessment methodology has been reported previously (Li et al., 2020). In the present study, executive function was particularly examined by the Controlled Oral Word Association Test (FAS) and Trail Making Test (TMT) B. All the raw component test scores were transformed to z scores. The signs of the z scores of TMT A and TMT B were reversed so that greater positive scores represented better performance. Domain composite scores were calculated by averaging the z scores of the component tests. The global cognition score was calculated by averaging all the composite domain scores. Paired *t*-test was then performed in the cognitive scores to compare changes between baseline and the 4-year follow-up.

### Image Acquisition

All the fMRI data were acquired on a Philips 3T Achieva Quasar Dual scanner (Philips Medical Systems, Best, Netherlands) at Neuroscience Research Australia (NeuRA, Sydney, New South Wales, Australia. A T2-weighted echo-planar imaging (EPI) sequence was used, with the following parameters: repetition time/echo time = 2,000/30 ms, flip angle = 90°, field of view = 240 × 130.5 × 240 mm^3^, continuous axial slices = 29, slice thickness = 4.5 mm without interslice gap, matrix size = 128 × 128, resulting in voxel size = 1.9 × 1.9 × 4.5 mm^3^. The participants were instructed to hold still, keep their eyes closed and think of nothing during the 7-min resting-state fMRI scans. 208 volumes were required per subject.

### Image Preprocessing

All the fMRI images were preprocessed and analyzed using a combination of DPABI^1^ (Yan et al., 2016) and Gretna^2^ (Wang et al., 2015), which are both toolboxes for data processing based on Statistical Parametric Mapping 12 (SPM12)^3^ (Ashburner et al., 2014). The images were corrected for slice timing to the median reference slice and were realigned for head motion correction after discarding the first 10 volumes. All the images were then normalized into Montreal Neurological Institute (MNI) space using an EPI template and were resliced with a voxel size of 3 mm × 3 mm × 3 mm to agree with the gray matter probability maps. The resultant images were smoothed with a 4-mm full-width half-maximum (FWHM) Gaussian Kernel and were bandpass filtered with a cut-off frequency of 0.01–0.1 Hz finally.

After quality control, three subjects were excluded under a head motion criterion of 3 mm and 3°; a total of 37 participants were ultimately included in this study.

### Functional Connectivity Analysis: Degree Centrality and Group Independent Component Analysis

To examine aging-related changes in functional connectivity of the brain, we first generated a whole-brain voxel-based degree map and explored the changes during the 4-year aging process (Li et al., 2020). For each voxel within the gray matter (cerebellum excluded), a temporal correlation of blood oxygen level-dependent (BOLD) signal time series with the left of the brain was evaluated using a Pearson’s correlation coefficient. Correlation coefficients above a threshold of *r* > 0.2 (Liao et al., 2013; Li et al., 2020) were added together to form the degree centrality of each voxel. This threshold was chosen to eliminate the low temporal correlation attributable to signal noise and also because the physiological basis of the negative correlations was ambiguous (Fox et al., 2009). The degree values were then converted to normally distributed z scores using the Fisher transformation to allow comparisons across subjects. Finally, the statistical significance of the differences in whole brain between the baseline and the 4-year follow-up were examined with paired *t*-test (10,000 random permutations, FWE-corrected: *p* ≤ 0.05). The permutation test was performed using Statistical Non-Parametric Mapping (SnPM) (Nichols and Holmes, 2014), a toolbox for SPM.

A group independent component analysis (GICA) (Rachakonda et al., 2007) was then applied to identify resting-state networks. It is a data-driven method of blind source signal separation which allows the extraction of signals of interest and not of interest without any prior information about the task. It could reveal characteristics of brain function and is thus widely used to extract resting-state brain networks. Preprocessed time series in all the participants at both time points were concatenated into one group, decomposed into group-independent time-courses, and back-reconstructed into voxel-based *z*-value spatial maps. The ICA analysis in this study was performed with a GIFT^4^ (Rachakonda et al., 2007) using a Fast ICA algorithm, and the data were decomposed into 30 components. Referring to ICA maps of resting-state networks and regions of interest of the selected networks from previous studies (Smith et al., 2009; Almgren et al., 2020), the spatial maps of networks of interest were thresholded into specific clusters, and the peak coordinates for regions were then obtained. These resultant sub-regions of specified networks were regarded as regions of interest in this study.

The FCS differences were re-examined using a paired *t*-test between the spatial maps of networks of interest for each participant at baseline and 4-year follow-up. A two-tailed Gaussian random field (GRF) theory correction (voxel-wise: minimum *z*-value > 3.29; cluster significance: *p* < 0.05) were applied for multiple comparisons.

### Generalized Linear Model for Time Series Extraction

The time series of the regions of interest were extracted by specifying and estimating a generalized linear model (GLM) using SPM. The method and parameters used here closely resemble those described in previous studies (Almgren et al., 2018, 2020) and contained a discrete cosine basis set to model resting-state activity (0.01–0.1 Hz) and 9 nuisance regressors that included 6 regressors for head motion and 3 regressors for the cerebrospinal fluid (CSF), white matter (WM), and global signals. The regressors of head motion (three translational, three rotational) were calculated during realignment. The CSF and WM signals were extracted with masks provided by the software package Gretna. Within the sphere (radius = 10 mm) centered on the coordinates determined by ICA, an F-contrast was specified across all the discrete cosine transform (DCT) components to test for high-amplitude fluctuations in brain activity in the preset frequency band. The peak voxel of the contrast map was then used as new coordinates of the ROI for each participant. The principal eigen-variate of voxels within the sphere (radius = 8 mm) centered on the new coordinates was summarized as the time series of ROI.

### Effective Connectivity: Definition of Models and Bayesian Model Selection

Dynamic causal modeling (DCM) (Friston et al., 2003) is a method developed specifically for neuroimage data analysis that explores the causal relations between brain areas. The neurodynamic component of DCM describes the interaction of neuronal activities, and the hemodynamic component models the transformation relationship from neuronal activity to blood oxygen-dependent level signals. Spectral DCM (spDCM) (Friston et al., 2014) extends the classical DCM method to the frequency domain, making it useful for the causal modeling of resting-state fMRI data. DCM is a model-based analysis that runs over predefined model space describing possible patterns of how these areas interact with each other based on prior knowledge. In this study, spDCM was performed on the models outlined to compare competing models. Analyses were performed with the DCM 12.5 module in SPM, with models parameterized using the bilinear differential equation for fMRI (Ashburner et al., 2014). For more detailed information about DCM, please see Supplementary Method.

**FIGURE 2:**
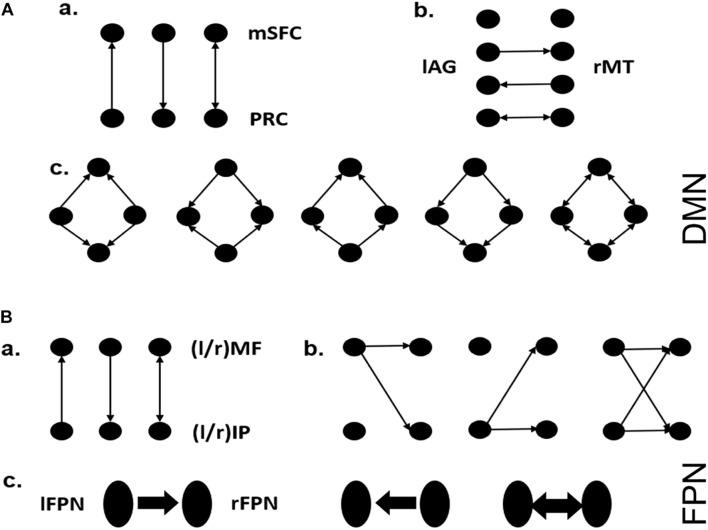
Model hypothesis for DMN and FPN. **(A)** a: Possible interaction pathways between mSFC and PRC, assuming they are directly connected. b: Possible interaction pathways between lAG and rMT. c: The symmetrical connections between the four brain regions, except for the connections involved in a and b. These assumptions yielded 60 possible dynamic causal modeling structures. **(B)** a: Possible interaction pathways between lMF and lIP (or rMF and rIP), assuming they are directly connected in each side of the FPN. b: The way one side of the FPN controls the other side via one or both nodes. c: The directionality by which one side of the FPN controls the other side via left to right or right to left or both directions. These assumptions yielded 81 possible dynamic causal modeling structures.

For a network with four nodes (e.g., DMN in this study), its full-connect model contains effective connections from each node to three other nodes and self-connections. Although the self-connection is usually considered to exist, there are still 2^12^ possible models in total. To reduce the model space to a reasonable range, in this work, we defined 3 model families for each of DMN and FPN (Figure 2; Di and Biswal, 2014). Each of the model families investigates a different pattern about how one brain area influences another or how one side influences the other side. For SMN, the existence of each interaction pathway was considered, and all 2^6^ possible model structures were estimated. Bayesian model selection (BMS) (Penny et al., 2010; Rigoux et al., 2014) based on random effect was then employed to determine the optimal model structure at the group level by investigating how interactions at the neuronal level may have generated the observed data. This method can appropriately reflect the requirements for the best compromise between model fitting accuracy and model complexity. BMS was performed on all the models in the model family of each network, and the exceedance probability, which is the probability that a certain model is more likely than any other model in the model space, was used to select a winning model for each model family. By using BMS at two time points, we took all the subjects as a group to explore optimal model changes that occur with aging. Specifically, the subjects were further divided into two subgroups based on the average global cognition level at two time points to compare the differences in the model structures and change patterns of elderly people with different cognition levels. No significant difference was found between demographic variables of groups divided based on global cognitive level (Supplementary Table 1).

## Results

### Neuropsychological Tests

The analyzed sample included 37 participants (16 males, 21 females; aged 78.65 ± 3.56 years at baseline; years of education 12.52 ± 2.81 at baseline). Only one of the participants received another 0.5 years of education during the follow-up period. The neuropsychological results are summarized in Table 1. The participants were found to have significant declines in processing speed, executive function and global cognition during the 4-year follow-up period.

**TABLE 1:** Cognitive domain scores.

Cognitive domain	Baseline	4-Year follow-up	*P*-value
Attention/processing speed	0.17 ± 1.21	−0.22 ± 1.21	0.033*
Language	−0.02 ± 0.99	0.03 ± 1.03	0.648
Executive function	0.35 ± 0.95	−0.02 ± 1.04	0.001*
Visuo-spatial	0.20 ± 0.99	0.06 ± 1.01	0.248
Memory	0.45 ± 0.98	0.35 ± 0.97	0.597
Global cognition	0.31 ± 0.99	0.06 ± 1.09	0.006*
MMSE	29.19 ± 0.97	29.43 ± 0.83	0.203

P-values were calculated with two-tailed paired t-tests between subjects at baseline and at the 4-year follow-up. Significant declines were found in attention/processing speed, executive function and global cognition during the 4-year follow-up. *Indicates a significant difference between two time points; MMSE = Mini-mental state examination.

### Functional Connectivity With Aging

**FIGURE 3:**
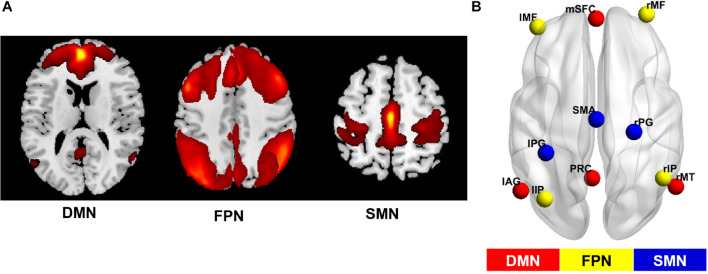
Networks of interest and their simplified sub-regions identified by GICA. **(A)** The spatial maps of the default mode network (DMN), the fronto-parietal network (FPN) and the somatomotor network (SMN) identified by group independent component analysis (GICA). The first two maps were thresholded with T = 1, while the third was thresholded with T = 3. Specifically, the FPN was synthesized from the left and right FPN components. **(B)** Locations of brain areas adopted from GICA results. The former networks were simplified as several main sub-regions, and the coordinates of peak values in the GICA map were chosen as center coordinates of the regions at the group level. This information was used to construct the dynamic causal model for each network. mSFC, medial superior frontal cortex; PRC, precuneus; lAG, left angular gyrus; rMT, right middle temporal gyrus; l/rMF, left/right middle frontal gyrus; l/rIP, left/right inferior parietal gyrus; SMA, supplementary motor area; l/rPG, left/right postcentral gyrus.

Comparing the degree centrality maps of all the participants at baseline and the 4-year follow-up, we found increased FCS in the SMA and the right medial temporal lobe and decreased FCS in the right superior parietal lobule (FWE-corrected *p* < 0.05) (Table 2 and Supplementary Figure 1). Using GICA, we were able to identify several important resting-state networks, and we determined that the networks that contained the functionally changed brain regions were the DMN, the FPN and the SMN (Figure 3A).

**TABLE 2:** Clusters showing significant functional connectivity changes with aging.

	Brain regions	Peak MNI coordinates, xyz	BA	*T*-value
Increased	SMA	0 –3 72	6	6.10
FCS	Right medial temporal lobe	57 –45 –6	21	4.15
Decreased FCS	Right superior parietal lobule	21 –60 60	7	4.92

*The clusters survived correction for family-wise error of p < 0.05. MNI, Montreal Neurological Institute; BA, Brodmann’s area; FCS, functional connectivity strength; SMA, supplementary motor area.*

A paired *t*-test on the GICA spatial maps of these networks between two time points reconfirmed the significant FCS changes in all three networks (Supplementary Figure 2). The main sub-regions of these networks and their peak MNI coordinates in the ICA map are listed in Supplementary Table 2, and their locations in the brain are shown in Figure 3B. These results were regarded as final regions and coordinates used to specify effective connectivity models.

### Bayesian Model Selection and Altered Propagation Pathway

**FIGURE 4:**
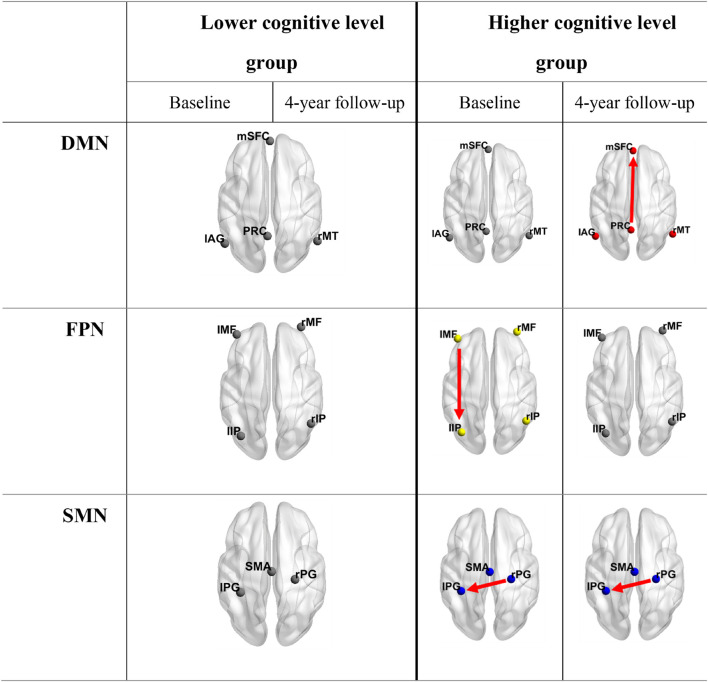
Optimal models for groups with different cognition levels at both time points. Connections shown in the table are lost information propagation pathways compared to fully connected models. It is obvious that the lower cognitive level group invariably had fully connected models. DMN, default mode network; FPN, fronto-parietal network; SMN, somatomotor network; mSFC, medial superior frontal cortex; PRC, precuneus; lAG, left angular gyrus; rMT, right middle temporal gyrus; l/rMF, left/right middle frontal gyrus; l/rIP, left/right inferior parietal gyrus; SMA, supplementary motor area; l/rPG = left/right postcentral gyrus.

Balancing the accuracy and complexity of models, BMS over the whole group revealed that all the networks at both time points, except the FPN at baseline, had full effective connect models (Supplementary Figure 3). The propagation from the left inferior parietal lobule to the left middle frontal gyrus in FPN was missed at baseline. When dividing the subjects into two groups according to global cognition level and performing the BMS again, the group with lower global cognition level (*N* = 19) invariably showed full-connect models. Although there was no uniform pattern of connection alteration in the group with higher cognition level (*N* = 18), missed connections were only observed in this group at both time points. The optimal models selected for each network at different conditions are shown in Figure 4 and Supplementary Table 3.

### Effective Connectivity Strength

**FIGURE 5:**
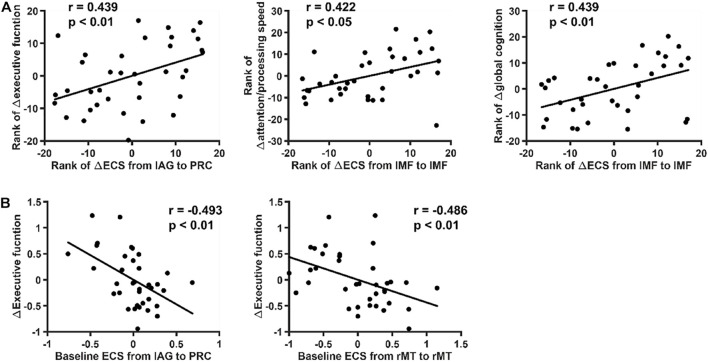
Relation between effective connectivity strength (ECS) and cognition. The relation between ECS and significantly declined cognition was explored. The data shown in the figure were controlled for sex, baseline age and education years. These *p*-values survived correction for FDR < 0.05. **(A)** Spearman correlation coefficients between 4-year changes of ECS in each network and significantly declined cognitive scores. Significant correlations were found between changes of ECS of self-inhibition of the left middle frontal gyrus and cognition alterations in attention/processing speed and global cognition. **(B)** Linear regression correlation coefficients between baseline ECS and cognitive decline. The ECS from the left angular gyrus to the precuneus was found to be predictive of executive function decline.

The Spearman coefficient was used to investigate the correlation between 4-year changes in ECS in each network and significantly declined cognitive scores, including scores associated with attention/processing speed, executive function, and global cognition (Figure 5A). Correlations were observed between changes in ECS of self-inhibition of the left middle frontal gyrus and cognition alterations in attention/processing speed (*r* = 0.422, *p* < 0.05) and global cognition (*r* = 0.439, *p* < 0.01). The ECS from the left angular gyrus to the precuneus was correlated with executive function (*r* = 0.439, *p* < 0.01). Linear regression was further performed to determine correlations between baseline ECS and cognition change to determine to what degree the cognition changes can be predicted (Figure 5B). In DMN, the strength of the flow of information initiating from the left angular gyrus to the precuneus and the strength of self-inhibition of the right middle temporal cortex were found to be predictive of executive function (the former: *r* = –0.493, *p* < 0.01; the latter: *r* = –0.486, *p* < 0.01). Sex, baseline age and education years were used as covariates in these analyses to better explore the role of ECS alterations in aging-related cognitive decline. These results were corrected for a false discovery rate (FDR) of *p* < 0.05. All the correlation results are shown in Supplementary Tables 4, 5.

## Discussion

In this longitudinal study of cognitively normal elderly people, we investigated the functionally changed brain regions and their networks and explored the patterns of change in the directionality and strength of causal interactions between intra- network areas. Our findings primarily provide information about two aspects. In terms of the directionality of effective connectivity, we found that the greater number of interaction pathways intra-networks is related to aging and cognitive decline, which might act as a compensatory mechanism, meaning that elderly individuals with lower cognitive levels would change to have full-connect networks earlier. In terms of the strength of effective connectivity, we showed that the flow of information from the left angular gyrus to the precuneus, which may facilitate the activation of memory retrieval to the functional hub involved in various cognitive domains (Gottlieb, 2007; Utevsky et al., 2014), was associated with aging-related declines in executive function. With the whole-brain FCS and ECS analyses, this study has expanded our understanding of the alterations in effective connectivity with aging and cognition decline.

The number of effective connections among networks was found to increase in the FPN with the aging process and was higher in the elderly people with lower cognition levels; full-connect models were recognized as optimal models in most cases. A possible explanation is that the increased number of effective connections is related to aging and cognitive decline. This suggests that elderly people with lower cognition levels may need more interaction pathways between brain areas to fulfill normal functions. In other words, elderly people with lower cognitive levels would change to have mutual interactions between all intra-network nodes earlier than elderly people with higher cognitive levels. Previous studies focused on elderly populations revealed that brain function dedifferentiates with aging, which is manifested by reduced selectivity in posterior regions and increased frontal recruitment across multiple tasks (Reuter-Lorenz et al., 2000; Davis et al., 2008; Goh, 2011). In fact, both aging-related increase and diminished functional connectivity have been reported in different regions, networks and ages (Damoiseaux et al., 2008; Biswal et al., 2010; Ferreira and Busatto, 2013). Moreover, the global degree of synchrony in functional activity between corresponding interhemispheric regions was found to decrease over the course of childhood and adolescence but to increase later in life (Zuo et al., 2010). And the increased frontal activation with age is a marker of an adaptive brain that engages in compensatory scaffolding in response to the challenges posed by declining neural structures and function (Park and Reuter-Lorenz, 2009). These studies showed that aging-related alterations in connectivity do not simply follow the same pattern. Despite the unclear nature of age-related functional changes, a reasonable interpretation for this observation is as follows. The functional decline of regions or information transmission pathways that perform specific functions may trigger compensatory mechanisms by engaging more alternative neural circuits to achieve a particular cognitive goal. These explain our findings that effective connections generally increase with aging and cognitive decline, especially in the FPN, the left middle frontal gyrus gained a connection toward the left inferior parietal gyrus with aging. And in the SMN, an extra interhemispheric connection was found in the subgroup with lower global cognition. Furthermore, in accordance with our results, recent studies in elderly people also observed increased effective connectivity compared to that in young people in networks involved in intelligible speech, retrieval, and motor execution (Cansino et al., 2017; Wang et al., 2019; Fei et al., 2020). Thus, it is not surprising that fully connected models appeared in most cases, considering the age of our participants. With this longitudinal study on the relationship between cognitive decline and effective connectivity, our findings provide reasonable evidence for compensatory mechanisms and support our views on aging and cognitive decline.

The change in self-inhibition connection strength of the left middle frontal gyrus in the FPN exhibited correlations with attention processing speed and global cognition decline. These results are consistent with previous studies describing the frontal lobe, whose gray matter volume decreases with aging (Jernigan et al., 2001) and is involved in a wide range of cognitive domains, including working memory, attention, and inhibition (Braver and Barch, 2002). It may seem contradictory that compared to the left side, the right middle frontal gyrus was proposed to be a link of the ventral and dorsal systems and to play an important role in the reorienting of attention (Corbetta et al., 2008; Japee et al., 2015). However, other studies focused on elderly people found that age-related changes in attentional processing are associated with the structure and functional characteristics of the left middle frontal cortex (Thomsen et al., 2004; Andersson et al., 2009). Thus, while the right middle frontal gyrus may be more important in general, the left is more relevant to aging-related cognitive decline. It is possible that this relevance only involves the left middle frontal gyrus itself, not its communication with other brain areas.

Interestingly, in the DMN, our results showed that the ECS from the left angular gyrus to the precuneus is predictive of the decline in executive function. In other words, the stronger the activation effect of the left angular gyrus on the precuneus is, the greater the degree of executive function decline will be. Similar predictability was also observed in the self-inhibition connection of the right middle temporal gyrus, which was proven to play a role in phonological discrimination (Xu et al., 2019). In the DMN, it was reported that the precuneus is a functional core involved in sensorimotor, cognition, and visual functions and has widespread anatomical connectivity with cortical and subcortical structures (Cavanna and Trimble, 2006; Margulies et al., 2009; Utevsky et al., 2014). However, the angular gyrus has been shown to be involved in the reorienting or shifting of attention, memory retrieval, and conflict resolution (Gottlieb, 2007; Seghier, 2013). These findings are consistent with our results since the executive function is measured by a combination of FAS and TMT B concerning word retrieval and complex attention, among other metrics. In addition, a recent study using DCM also found that memory retrieval is a weak modulation on the angular gyrus-precuneus connection (Ren et al., 2018). Together, these findings provide a possible explanation that the decline in executive function during the aging process is mainly caused by decreased memory retrieval and attention reorientation ability. More importantly, the strength of flow of information from the left angular gyrus to the precuneus, which is associated with activation of memory retrieval and the functional hub involved in various cognitive domains, may serve as a predictor for the extent of the aging-related decline in executive function. This may provide clinicians with new perspectives for interventions, for example, take complementary memory retrieval training as part of executive rehabilitation. Moreover, in therapeutic interventions like transcranial magnetic stimulation, the left angular gyrus would be a better target than the precuneus, despite it is the interaction between both areas that may matter.

With regard to the research methods, five limitations need to be acknowledged. First, considering the complexity of the model space, only major clusters derived from FCS analysis were included as the regions of interest intra-network. Additionally, the model space was further simplified based on previous studies and focused on the connection patterns we wanted to explore. Connections and areas beyond this were not modeled and discussed. Second, although in most cases, the exceedance probabilities of the optimal models are well above others, some are not (Supplementary Table 3). However, these more equivocal model selection results should not affect the outcomes of the study since only the contrast of fully and non-fully connected models in subjects at different time points and in subjects with different cognitive levels were involved in the inference. This may reflect the heterogeneity among subjects, despite having approximately the same trends of change. Third, our sample size was modest for a longitudinal study of normal elderly. But given the participants were very old (aged 70–90 at baseline) and the critical “cognitively normal” inclusion criteria, this study can bring new insights into longitudinal changes of normal aging in the old-aged group. Fourth, the lack of multimodal data to better model the causality between brain areas and further investigate the mechanism is another limitation of this study. Also, only one follow-up point data was obtained. Longer follow-up period—another follow-up point would help to better picture the aging process and validate the change patterns of brain connectivity and its interactions with cognition. As the MAS research continues and the time span increases, this would be well addressed.

While the dataset used in this study is longitudinal and targeting at the normal elderly (aged 70–90), the Cam-CAN dataset is cross-sectional adult-lifespan (aged 18–87) population-based (Taylor et al., 2017). With the high spatial resolution of fMRI and high temporal resolution of EEG, applying causal analysis on the Cam-CAN dataset should be a promising prospect. With more available multimodal data, the findings like alterations of numbers of interaction pathways and the practicality and accuracy of predictors might be better validated then.

Generally, our findings indicated that the inner networks of the interaction pathways increase with aging and cognitive decline and that the propagation from the left angular gyrus to the precuneus may be a key factor underlying declines in executive function.

## Data Availability Statement

The data analyzed in this study is subject to the following licenses/restrictions: The dataset used and analyzed are available to other researchers subject to review of the request by the Scientific Committee of the study and ethics approval. Requests to access these datasets should be directed to the corresponding author.

## Ethics Statement

The studies involving human participants were reviewed and approved by the Ethics Committees of the University of New South Wales and the South Eastern Sydney and Illawarra Area Health Service. The patients/participants provided their written informed consent to participate in this study.

## Author Contributions

XC: drafting and revision of the manuscript for content, analysis, and interpretation of data. TL: study concept and design, interpretation of data. JJ: discussion of study. HL, JZ, and HN: analysis of data. NK, HB, and PS: acquisition and interpretation of data. WW: interpretation of data, critical revision of the manuscript for intellectual content. All authors participated in manuscript revision and final approval.

## Conflict of Interest

The authors declare that the research was conducted in the absence of any commercial or financial relationships that could be construed as a potential conflict of interest.

## Publisher’s Note

All claims expressed in this article are solely those of the authors and do not necessarily represent those of their affiliated organizations, or those of the publisher, the editors and the reviewers. Any product that may be evaluated in this article, or claim that may be made by its manufacturer, is not guaranteed or endorsed by the publisher.

## Footnotes

1
http://www.rfmri.org/dpabi/

2
https://www.nitrc.org/projects/gretna/

3
https://www.fil.ion.ucl.ac.uk/spm/software/spm12/

4
https://trendscenter.org/software/gift/
