# Supplementary material for: Alternation in Effective Connectivity With Cognitive Aging: A Longitudinal Study of Elderly Populations
Source: Front Aging Neurosci. 2021 Nov 12;13:755931. doi: 10.3389/fnagi.2021.755931 (PMC8636113; doi:10.3389/fnagi.2021.755931)
Supplement: Supplementary file 1 [file Data_Sheet_1.docx]

Supplementary Method

# DCM

Dynamic causal modeling (DCM) is a method developed specifically for neuroimage data analysis(K. J. Friston, Harrison, & Penny, 2003). DCM for fMRI consists of two parts: (1) the neuronal state model, describing how the dynamics of coupled neuronal populations interact, and (2) the hemodynamic model, which transforms hidden neural states of each population or region into predicted BOLD responses. For resting state activity, the neural state equation (Figure 1) describes the nature of the three components which underlie the modeled neural dynamics: (i) context-independent effective connectivity among brain regions, mediated by anatomical connections (A), (ii) direct inputs into the system that drive regional activity (C), (iii) the state noise (resp. the measurement or observation noise) – modelling the random neuronal fluctuations that drive the resting state activity(v)(Ashburner et al., 2014). With the hemodynamic model, the hidden state is observable by fMRI. The biophysical parameters of the hemodynamic model are shown in Table 1.


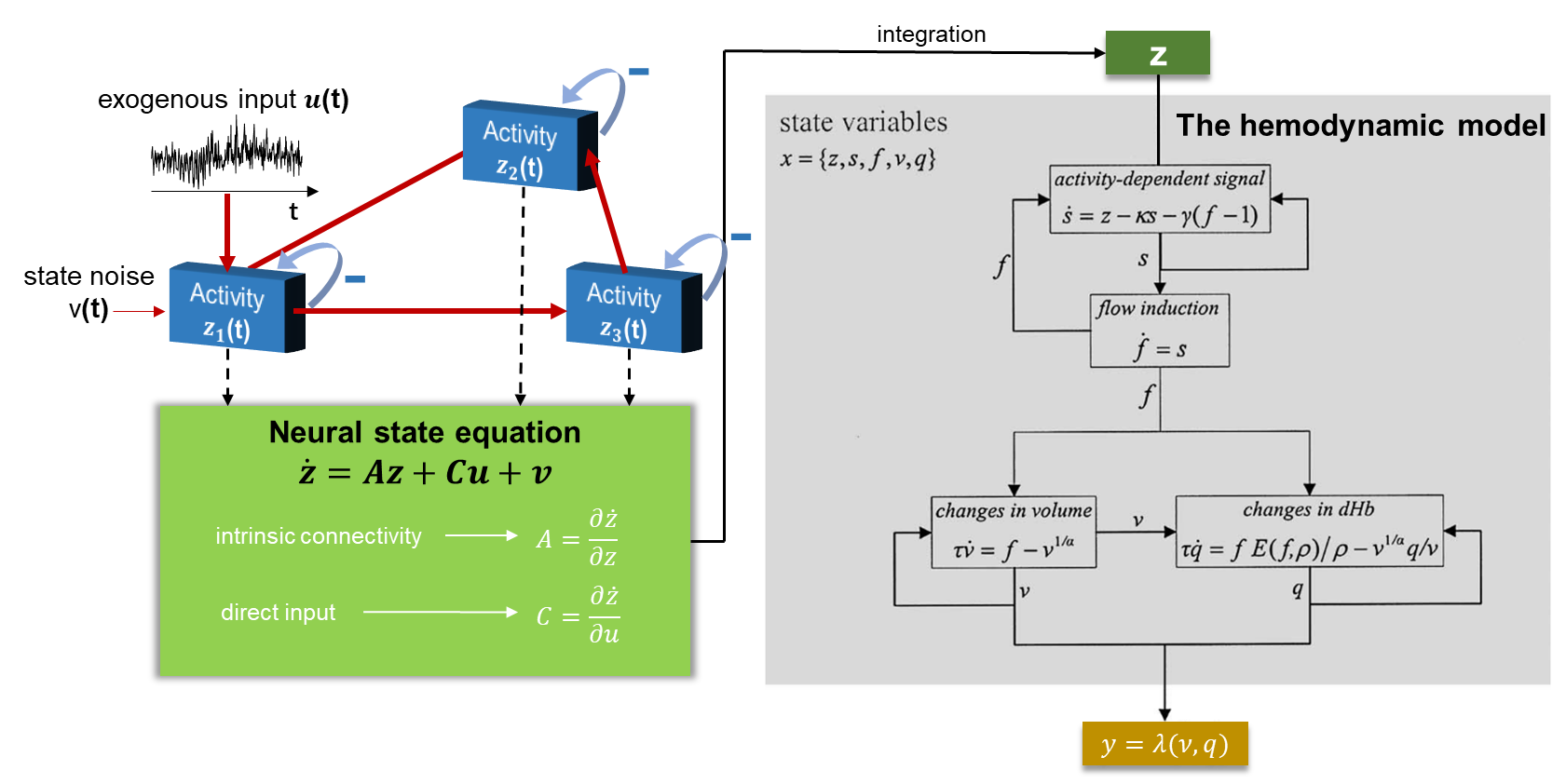


**Figure 1.** Schematic summary of the conceptual basis of DCM for resting state fMRI. (Left) The dynamics in a system of interacting neuronal populations (blue boxes), which are not directly observable by fMRI, is modeled using a bilinear state equation (grey box). Integrating the state equation gives predicted neural dynamics (z) that enter a model of the hemodynamic response (λ) to give predicted BOLD responses (y) (brown box). The parameters at both neural and hemodynamic levels are adjusted such that the differences between predicted and measured BOLD series are minimized. We can include exogenous (or experimental) inputs, u in the model since it is perfectly possible to have external, (non-modulatory) stimuli in conventional functional neuroimaging studies. These inputs drive the hidden states – and are usually set to zero in resting state models. A is the Jacobian describing the behavior – i.e. the effective connectivity – of the system near its stationary point in the absence of the fluctuations v. (Right) Neuronal activity induces a vasodilatory and activity-dependent signal s that increases the flow $f$. Flow causes changes in volume and deoxyhemoglobin (v and q). These two hemodynamic states enter the output nonlinearity to give the observed BOLD response y. This transformation from neuronal states $z_{i}$ to hemodynamic response $y_{i}$ is encoded graphically by the dark grey boxes.(Ashburner et al., 2014; K. J. Friston et al., 2003)

| Parameter | Description | Prior mean | Prior Variance |
| --- | --- | --- | --- |
| $\boldsymbol{\kappa}$ | Rate of signal decay | 0.65 per s | 0.015 |
| $\boldsymbol{\gamma}$ | Rate of flow-dependent elimination | 0.41 per s | 0.002 |
| $\boldsymbol{\tau}$ | Hemodynamic transit time | 0.98 s | 0.0568 |
| $\boldsymbol{\alpha}$ | Grubb’s exponent | 0.32 | 0.0015 |
| $\boldsymbol{\rho}$ | Resting oxygen extraction fraction | 0.34 | 0.024 |

**Table 1.** Priors on biophysical parameters

Spectral DCM (spDCM)(Karl J Friston, Kahan, Biswal, & Razi, 2014) extends the classical DCM method from the time domain to the frequency domain, making it useful for the causal modeling of resting state fMRI data. It uses a neuronally plausible power-law model of the coupled dynamics of neuronal populations to generate complex cross spectra among measured responses. The stochastic generative model is written as:

$$\begin{aligned} &\dot{x}(t)=f(x,\theta)+v(t) \\ &y(t)=g(x,\theta)+e(t) \end{aligned}$$

$\begin{aligned} &\left\langle v(t)\cdot v(t-\tau)^{T} \right)=\rho_{v}(\tau,\theta)=F^{-1}\left( g_{v}(\omega,\theta) \right) \\ &\left\langle\varepsilon(t)\cdot\varepsilon(t-\tau)^{T} \right)=\rho_{\varepsilon}(\tau,\theta)=F^{-1}\left( g_{\varepsilon}(\omega,\theta) \right) \end{aligned}$(Eq 1)

$$\theta\supseteq\{A,C,\alpha,\beta\}$$

It comprises differential equations coupling hidden states (first line) and an observer equation mapping hidden states x(t) to observed responses y(t) (second line). Crucially, both the motion of hidden states and responses are subject to random fluctuations, also known as state v(t) and observation e(t) noise. The form of these fluctuations are modelled in terms of their cross covariance functions ρ(t) of time t or cross spectral density functions g(ω) of radial frequency ω, as shown in the lower equations. Given this state space model and its parameters θ (which include effective connectivity) one can now parameterize a series of representations of statistical dependencies among successive responses as shown in the second row. The mapping between these representations rests on the Fourier transform, denoted by F and its inverse. The priors for θ used in DCM for fMRI are listed in Table 2.

| Parameter | Description | Prior mean | Prior Variance |
| --- | --- | --- | --- |
| $\mathbf{ln(-}\boldsymbol{A}_{\boldsymbol{ii}}\mathbf{)}$ | Inhibitory self connections | $\ln\frac{1}{2}$ | $\frac{1}{256}$ |
| $\boldsymbol{A}_{\boldsymbol{ij}}$ | Extrinsic effective connectivity | $\frac{1}{128}$ | $\frac{1}{64}$ |
| $\mathbf{C}$ | Exogenous input scaling | 0 | 1 |
| $\boldsymbol{ln(\alpha)}$ | Amplitude of fluctuations | 0 | $\frac{1}{64}$ |
| $\boldsymbol{ln(\beta)}$ | Exponent offluctuations | 0 | $\frac{1}{64}$ |

**Table 2**. Priors on parameters in spDCM(Karl J Friston et al., 2014)

In the current paper, the DCM analyses were performed with the DCM 12.5 module in SPM.

Supplementary Tables and Figures

# Supplementary Tables

| Variable | Low cognition | High cognition | P value |
| --- | --- | --- | --- |
| Age (mean±SD, years) | 79.22±3.77 | 78.04±3.33 | 0.3172 |
| Sex (male=1, female=2) | 1.68±0.48 | 1.44±0.51 | 0.1492 |
| Education (mean±SD, years) | 11.66±3.09 | 13.43±2.21 | 0.0538 |

**Table 1.** Demographic characteristics of groups divided based on global cognitive level.

| Network name | Regions of interest | ROI abbreviation | MNI coordinates, xyz |
| --- | --- | --- | --- |
| DMN  (default mode network) | Precuneus | PRC | -3 -57 30 |
|  | Medial superior frontal cortex | mSFC | 0 57 12 |
|  | Left angular gyrus | lAG | -54 -66 33 |
|  | Right middle temporal gyrus | rMT | 57 -63 24 |
| FPN  (fronto-parietal network) | Left middle frontal gyrus | lMF | -42 51 -6 |
|  | Right middle frontal gyrus | rMF | 36 60 3 |
|  | Left inferior parietal gyrus | lIP | -37 -72 51 |
|  | Right inferior parietal gyrus | rIP | 48 -57 54 |
| SMN  (somatomotor network) | Supplementary motor area | SMA | 0 -15 57 |
|  | Left postcentral gyrus | lPG | -36 -39 66 |
|  | Right precentral gyrus | rPG | 27 -24 69 |

**Table 2.** Networks and regions included in this study.

All the networks and regions included in this study and the MNI coordinates used for group level are listed in the table. ROI = regions of interest; MNI = Montreal Neurological Institute.

|  | **Default mode network** | | | |
| --- | --- | --- | --- | --- |
| Group | Baseline | | 4-year follow-up | |
| Model exceedance probability |  | |  | |
| Optimal model | Model 60 | | Model 60 | |
| Group | Low cognition level | High cognition level | Low cognition level | High cognition level |
| Model exceedance probability |  |  |  |  |
| Optimal model | Model 60 | Model 60 | Model 60 | Model 40 (PRC->mSFC) |
|  | ***Fronto-parietal network*** | | | |
| Group | Baseline | | 4-year follow-up | |
| Model exceedance probability |  | |  | |
| Optimal model | Model 79 (lIP->lMF) | | Model 81 | |
| Group | Low cognition level | High cognition level | Low cognition level | High cognition level |
| Model exceedance probability |  |  |  |  |
| Optimal model | Model 81 | Model 80 (lMF->lIP) | Model 81 | Model 81 |
|  | ***Somatomotor network*** | | | |
| Group | Baseline | | 4-year follow-up | |
| Model exceedance probability |  | |  | |
| Optimal model | Model 64 | | Model 64 | |
| Group | Low cognition level | High cognition level | Low cognition level | High cognition level |
| Model exceedance probability |  |  |  |  |
| Optimal model | Model 64 | Model 63 (rPG->lPG) | Model 64 | Model 63 (rPG->lPG) |

**Table 3.** The exceedance probability of the models obtained by Bayesian model selection.

As a quantitative measure of the amount of evidence for the optimal model, the exceedance probabilities were obtained by Bayesian model selection based on random effect are shown with bar graphs in the table. The numbers of the optimal models are listed below the bars. And the lost information propagation pathways compared to fully connected models are shown in brackets. PRC = precuneus; mSFC = medial superior frontal cortex; lIP = left inferior parietal gyrus; lMF = left middle frontal gyrus; l/rPG = left/right postcentral gyrus.

| DMN | | | |
| --- | --- | --- | --- |
| Effective Connection | Attention/Processing speed | Executive function | Global cognition |
| 1.PRC->PRC | -0.0283(0.8738) | 0.2059(0.2428) | 0.0155(0.9307) |
| 2.mSFC->PRC | 0.0411(0.8173) | -0.2049(0.2451) | -0.1144(0.5193) |
| 3.lAG->PRC | -0.1454(0.4121) | **0.4387(0.0094)** | 0.0092(0.9590) |
| 4.rMT->PRC | -0.2961(0.0890) | -0.1371(0.4394) | -0.2921(0.0936) |
| 5.PRC->mSFC | 0.1867(0.2904) | 0.2303(0.1901) | 0.0889(0.6172) |
| 6.mSFC->mSFC | 0.1170(0.5099) | 0.2740(0.1169) | 0.1050(0.5545) |
| 7.lAG->mSFC | 0.1119(0.5288) | -0.0217(0.9032) | -0.0583(0.7431) |
| 8.rMT->mSFC | 0.0345(0.8466) | 0.2392(0.1730) | 0.0044(0.9804) |
| 9.PRC->lAG | 0.0991(0.5772) | -0.0872(0.6240) | -0.0708(0.6906) |
| 10.mSFC->lAG | -0.0056(0.9751) | -0.0516(0.7721) | 0.0669(0.7070) |
| 11.lAG->lAG | 0.0713(0.6887) | -0.1995(0.2580) | 0.0353(0.8431) |
| 12.rMT->lAG | -0.2088(0.2359) | 0.0266(0.8811) | -0.1921(0.2765) |
| 13.PRC->rMT | 0.2425(0.1669) | -0.2839(0.1037) | 0.1451(0.4128) |
| 14.mSFC->rMT | -0.1255(0.4793) | -0.0899(0.6131) | -0.2782(0.1112) |
| 15.lAG->rMT | -0.0603(0.7348) | 0.3244(0.0613) | 0.1581(0.3718) |
| 16.rMT->rMT | -0.2659(0.1285) | 0.0724(0.6842) | -0.1258(0.4785) |
| FPN | | | |
| Effective Connection | Attention/Processing speed | Executive function | Global cognition |
| 1.lMF->lMF | **0.4217(0.0130)** | -0.0453(0.7993) | **0.4393(0.0093)** |
| 2.rMF->lMF | -0.1918(0.2771) | -0.2760(0.1141) | -0.2413(0.1692) |
| 3.lIP->lMF | — | — | — |
| 4.rIP->lMF | 0.2432(0.1658) | 0.2331(0.1846) | 0.2353(0.1805) |
| 5.lMF->rMF | 0.0273(0.8781) | 0.0219(0.9023) | -0.0851(0.6324) |
| 6.rMF->rMF | 0.0119(0.9469) | 0.0233(0.8959) | 0.0249(0.8886) |
| 7.lIP->rMF | 0.0789(0.6572) | -0.2422(0.1675) | 0.2197(0.2118) |
| 8.rIP->rMF | -0.2201(0.2111) | 0.1111(0.5317) | -0.3269(0.0591) |
| 9.lMF->lIP | 0.0789(0.6572) | 0.1240(0.4847) | -0.0499(0.7791) |
| 10.rMF->lIP | 0.0031(0.9861) | 0.1660(0.3482) | -0.0558(0.7538) |
| 11.lIP->lIP | 0.2124(0.2278) | 0.0256(0.8857) | 0.2546(0.1462) |
| 12.rIP->lIP | 0.0928(0.6015) | -0.2560(0.1439) | -0.0536(0.7634) |
| 13.lMF->rIP | 0.2611(0.1359) | 0.0309(0.8623) | 0.0850(0.6327) |
| 14.rMF->rIP | 0.0959(0.5896) | 0.0633(0.7223) | 0.2000(0.2569) |
| 15.lIP->rIP | 0.0917(0.6061) | -0.0059(0.9738) | 0.1195(0.5010) |
| 16.rIP->rIP | 0.0744(0.6757) | 0.0466(0.7935) | 0.1660(0.3481) |
| SMN | | | |
| Effective Connection | Attention/Processing speed | Executive function | Global cognition |
| 1.SMA->SMA | -0.2293(0.1921) | 0.2891(0.0973) | -0.1177(0.5073) |
| 2.lPG->SMA | -0.4074(0.0168) | -0.1482(0.4028) | -0.2439(0.1645) |
| 3.rPG->SMA | 0.1379(0.4369) | 0.2803(0.1083) | 0.0480(0.7875) |
| 4.SMA->lPG | 0.0089(0.9604) | -0.2718(0.1199) | -0.2139(0.2245) |
| 5.lPG->lPG | -0.3625(0.0351) | -0.2397(0.1722) | -0.3654(0.0336) |
| 6.rPG->lPG | -0.1385(0.4349) | -0.0439(0.8052) | -0.1363(0.4421) |
| 7.SMA->rPG | 0.1616(0.3612) | -0.3163(0.0684) | 0.0334(0.8512) |
| 8.lPG->rPG | -0.0888(0.6173) | 0.0063(0.9716) | -0.1511(0.3937) |
| 9.rPG->rPG | -0.2707(0.1215) | -0.2757(0.1146) | -0.2638(0.1316) |

**Table 4.** The correlation between 4-year changes in ECS in each network and cognitive scores.

| DMN | | | |
| --- | --- | --- | --- |
| Effective Connection | Attention/Processing speed | Executive function | Global cognition |
| 1.PRC->PRC | -0.0283(0.8738) | 0.2059(0.2428) | 0.0155(0.9307) |
| 2.mSFC->PRC | 0.0411(0.8173) | -0.2049(0.2451) | -0.1144(0.5193) |
| 3.lAG->PRC | -0.1454(0.4121) | 0.4387(0.0094) | 0.0092(0.9590) |
| 4.rMT->PRC | -0.2961(0.0890) | -0.1371(0.4394) | -0.2921(0.0936) |
| 5.PRC->mSFC | 0.1867(0.2904) | 0.2303(0.1901) | 0.0889(0.6172) |
| 6.mSFC->mSFC | 0.1170(0.5099) | 0.2740(0.1169) | 0.1050(0.5545) |
| 7.lAG->mSFC | 0.1119(0.5288) | -0.0217(0.9032) | -0.0583(0.7431) |
| 8.rMT->mSFC | 0.0345(0.8466) | 0.2392(0.1730) | 0.0044(0.9804) |
| 9.PRC->lAG | 0.0991(0.5772) | -0.0872(0.6240) | -0.0708(0.6906) |
| 10.mSFC->lAG | -0.0056(0.9751) | -0.0516(0.7721) | 0.0669(0.7070) |
| 11.lAG->lAG | 0.0713(0.6887) | -0.1995(0.2580) | 0.0353(0.8431) |
| 12.rMT->lAG | -0.2088(0.2359) | 0.0266(0.8811) | -0.1921(0.2765) |
| 13.PRC->rMT | 0.2425(0.1669) | -0.2839(0.1037) | 0.1451(0.4128) |
| 14.mSFC->rMT | -0.1255(0.4793) | -0.0899(0.6131) | -0.2782(0.1112) |
| 15.lAG->rMT | -0.0603(0.7348) | 0.3244(0.0613) | 0.1581(0.3718) |
| 16.rMT->rMT | -0.2659(0.1285) | 0.0724(0.6842) | -0.1258(0.4785) |
| FPN | | | |
| Effective Connection | Attention/Processing speed | Executive function | Global cognition |
| 1.lMF->lMF | 0.4217(0.0130) | -0.0453(0.7993) | 0.4393(0.0093) |
| 2.rMF->lMF | -0.1918(0.2771) | -0.2760(0.1141) | -0.2413(0.1692) |
| 3.lIP->lMF | — | — | — |
| 4.rIP->lMF | 0.2432(0.1658) | 0.2331(0.1846) | 0.2353(0.1805) |
| 5.lMF->rMF | 0.0273(0.8781) | 0.0219(0.9023) | -0.0851(0.6324) |
| 6.rMF->rMF | 0.0119(0.9469) | 0.0233(0.8959) | 0.0249(0.8886) |
| 7.lIP->rMF | 0.0789(0.6572) | -0.2422(0.1675) | 0.2197(0.2118) |
| 8.rIP->rMF | -0.2201(0.2111) | 0.1111(0.5317) | -0.3269(0.0591) |
| 9.lMF->lIP | 0.0789(0.6572) | 0.1240(0.4847) | -0.0499(0.7791) |
| 10.rMF->lIP | 0.0031(0.9861) | 0.1660(0.3482) | -0.0558(0.7538) |
| 11.lIP->lIP | 0.2124(0.2278) | 0.0256(0.8857) | 0.2546(0.1462) |
| 12.rIP->lIP | 0.0928(0.6015) | -0.2560(0.1439) | -0.0536(0.7634) |
| 13.lMF->rIP | 0.2611(0.1359) | 0.0309(0.8623) | 0.0850(0.6327) |
| 14.rMF->rIP | 0.0959(0.5896) | 0.0633(0.7223) | 0.2000(0.2569) |
| 15.lIP->rIP | 0.0917(0.6061) | -0.0059(0.9738) | 0.1195(0.5010) |
| 16.rIP->rIP | 0.0744(0.6757) | 0.0466(0.7935) | 0.1660(0.3481) |
| SMN | | | |
| Effective Connection | Attention/Processing speed | Executive function | Global cognition |
| 1.SMA->SMA | -0.2293(0.1921) | 0.2891(0.0973) | -0.1177(0.5073) |
| 2.lPG->SMA | -0.4074(0.0168) | -0.1482(0.4028) | -0.2439(0.1645) |
| 3.rPG->SMA | 0.1379(0.4369) | 0.2803(0.1083) | 0.0480(0.7875) |
| 4.SMA->lPG | 0.0089(0.9604) | -0.2718(0.1199) | -0.2139(0.2245) |
| 5.lPG->lPG | -0.3625(0.0351) | -0.2397(0.1722) | -0.3654(0.0336) |
| 6.rPG->lPG | -0.1385(0.4349) | -0.0439(0.8052) | -0.1363(0.4421) |
| 7.SMA->rPG | 0.1616(0.3612) | -0.3163(0.0684) | 0.0334(0.8512) |
| 8.lPG->rPG | -0.0888(0.6173) | 0.0063(0.9716) | -0.1511(0.3937) |
| 9.rPG->rPG | -0.2707(0.1215) | -0.2757(0.1146) | -0.2638(0.1316) |

**Table 5.** The correlation between baseline ECS in each network and cognition scores.

# Supplementary Figures


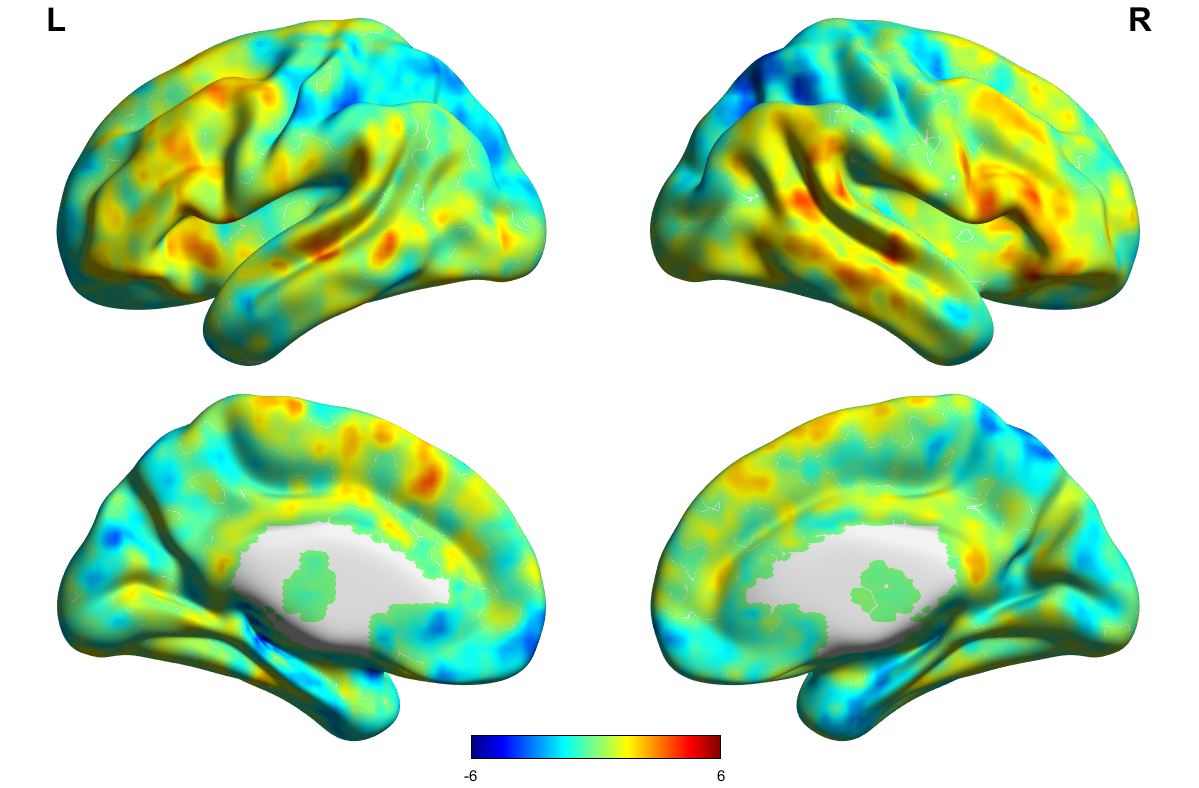


**Figure 1.** Changes in whole-brain voxel-based functional connectivity.

A paired t test was used to investigate the changes in whole-brain voxel-based functional connectivity (measured by degree map) with aging. Red indicates increased functional connectivity compared to baseline, while blue indicates the opposite. This figure shows the uncorrected results of paired t testing between functional connectivity at baseline and at the 4-year follow-up for a full view of functional change pattern. Corrected statistical results can be found in Table 3 in the article.


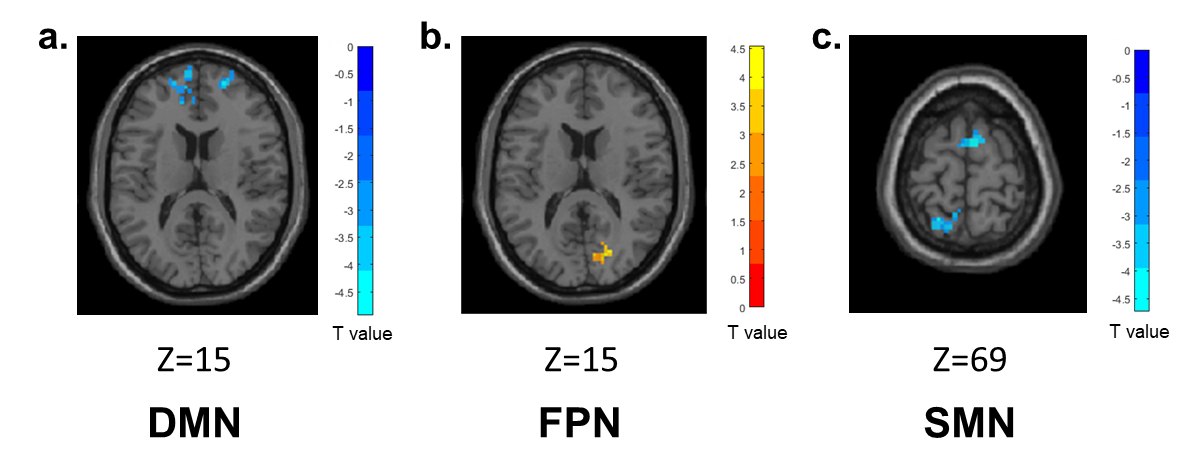


**Figure 2.** Functionally changed regions identified by GICA.

A paired t test between two time points was used to re-investigate the changes of functional connectivity inner networks (measured by spatial maps generated by GICA) with aging. Multiple comparisons were carried out by two-tailed Gaussian random field theory correction (voxel-wise: minimum z-value > 3.29; cluster significance: p < 0.05). Significant changes were found in each of the networks of interest selected in this study.


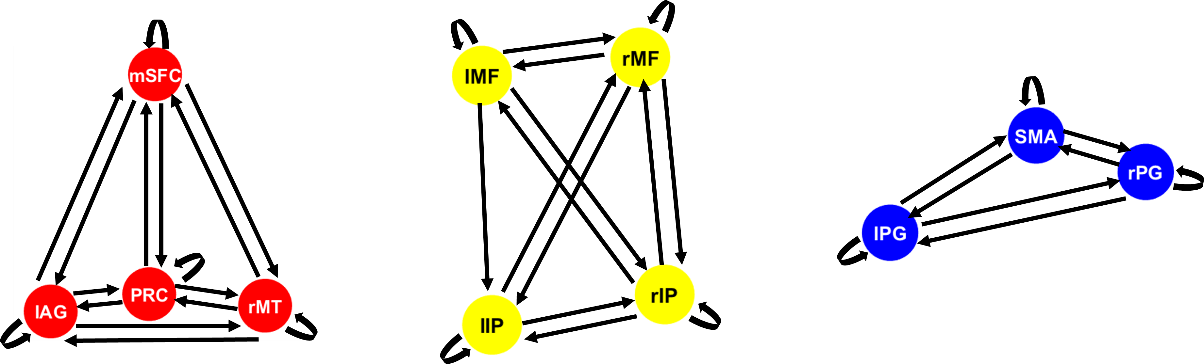


**Figure 3.** Optimal models selected at the group level for all participants at baseline.

Both the DMN and SMN had fully connected models at baseline, meaning that direct causal interactions exist between each pair of nodes in the inner networks. Only the FPN exhibited a lost connection from the left middle frontal gyrus to the left inferior parietal gyrus. At the 4-year follow-up, all the networks changed to have fully connected models. mSFC = medial superior frontal cortex; PRC = precuneus; lAG = left angular gyrus; rMT = right middle temporal gyrus; l/rMF = left/right middle frontal gyrus; l/rIP = left/right inferior parietal gyrus; SMA = supplementary motor area; l/rPG = left/right postcentral gyrus.

**References**

Ashburner, J., Barnes, G., Chen, C., Daunizeau, J., Flandin, G., Friston, K., . . . Moran, R. (2014). SPM12 manual. *Wellcome Trust Centre for Neuroimaging, London, UK*, 2464.

Friston, K. J., Harrison, L., & Penny, W. (2003). Dynamic causal modelling. *Neuroimage, 19*(4), 1273-1302. doi:10.1016/s1053-8119(03)00202-7

Friston, K. J., Kahan, J., Biswal, B., & Razi, A. (2014). A DCM for resting state fMRI. *Neuroimage, 94*, 396-407. doi:10.1016/j.neuroimage.2013.12.009
